# Labour trafficking: Challenges and opportunities from an occupational health perspective

**DOI:** 10.1371/journal.pmed.1002440

**Published:** 2017-11-22

**Authors:** Elena Ronda-Pérez, Bente E. Moen

**Affiliations:** 1 Public Health Department, University of Alicante, Alicante, Spain; 2 Centre for International Health, Department of Global Public Health and Primary Care, University of Bergen, Bergen, Norway

## Abstract

In this essay for the collection on Human Trafficking, Exploitation, and Health, Elena Ronda-Perez and colleague discuss ways occupational health services can detect and address labour trafficking.

**Summary points**

- Labour trafficking is intrinsically related to occupational health; however, very little attention has been paid to the issue from an occupational health perspective.
- The recognition of certain work-related health problems in workers in specific work sectors can help to identify victims of labour trafficking.
- This essay identifies a series of opportunities for occupational health services to detect and address labour trafficking and increase health personnel awareness of the problem.

## Labour trafficking as a health issue

Labour trafficking is a form of modern-day slavery in which individuals are recruited or transported to perform labour or services through the use of force, fraud, or coercion and under the threat of some kind of penalty. In addition, the person has not offered himself/herself voluntarily for the activity in question. The International Labour Organization (ILO) uses the expression ‘forced labour’ instead of the word ‘trafficking’ and estimates that 25 million people are victims of forced labour [1]. This represents about 5.4 out of every 1,000 people globally. Approximately 90% are exploited by private individuals and enterprises. While in the past the ILO focused narrowly on sex trafficking, recently more attention has been paid to a broader range of sectors and circumstances in which forced labour may take place [2]. Global assessments suggest that a substantial proportion of labour migrants end up in situations of extreme exploitation, some of which are formally identified as human trafficking [2,3]. These problems can occur in any country, though the relative magnitude of the problem in different countries is not clear. The workers involved represent both genders [4] and come from different geographic regions including Asia, Africa, Latin America, and Eastern Europe, in addition to other parts of the European Union [3].

Current approaches to addressing human trafficking have been developed through the work of Zimmerman [2] and Dowling [3]. Inclusion of a health perspective—and an occupational health perspective in particular—can contribute to addressing the problem. With additional knowledge and awareness of the relationship between work and health, health professionals will be better equipped to identify and treat specific health problems among trafficked workers. Development of appropriate policies and regulations within the health sector is also needed.

This article aims to describe the occupational health perspective and approach to addressing human trafficking.

## Implementing strategies from an occupational health perspective

The main task of the field of occupational health is to avoid and reduce the effect of workplace factors that can have an adverse impact on the health of workers. These factors can be physical, organizational, or psychosocial in nature. There are several definitions of occupational health, but the most commonly used definition is that developed by the Joint ILO/WHO Committee on Occupational Health (1950). Occupational health is defined as the ‘promotion and maintenance of the highest degree of physical, mental, and social well-being of workers in all occupations’ [5].

Many countries have specific occupational health services (OHSs) that employ health personnel to provide essential preventive services at workplaces to achieve the ILO’s goal. An OHS can be established within a company, or the services can be subcontracted from an external OHS. OHSs function differently in different countries, but in general, they play an advisory role related to the prevention of adverse health effects of the work environment. The aim of an OHS is to help employers and employees comply with local and organizational health and safety regulations. An OHS normally works in tandem with local unions and/or the labour inspectorate.

## Work sectors related to risk of labour trafficking and health consequences

Labour trafficking is seen in work sectors such as agriculture, factory work, fishing, mining, construction, hospitality industries, and domestic services [2,4,6,7] (Table 1). These work sectors are characterised by frequent labour shortages and use of subcontractors, often in the informal economy. Also, trafficking occurs mainly among migrant workers [3].

**Table 1:** Main sectors of work known to be related to labour trafficking and their possible health problems [2,4].

Work sector	Examples of work-related health problems that may put trafficked workers into contact with health systems
Agriculture	Injuries Pesticide intoxications Heat stress
Cleaning	Injuries
Construction	Injuries Heat stress, cold stress
Fishing	Injuries Heat stress, cold stress
Mining	Injuries Heat stress
Factory work: Textile industry Food processing Brick production Chemical production	Injuries Heat stress Acute intoxications (in chemical production)
Hospitality: Restaurants, food service Hotels	Acute tendonitis Muscular sprains
Domestic service: Cleaning in private homes	Acute tendonitis Muscular sprains

The risks and health problems associated with the different types of work in sectors commonly involved in trafficking are already quite well described in the occupational health literature but only for certain health problems in mainstream (i.e., non-trafficked) working populations and primarily in high-income countries.

Similar to what has been described in the literature on migrant workers [8], the situation of trafficked workers warrants exploration from an occupational health perspective because of the lack of available surveillance data on these workers and the inadequacy of standard occupational health protection mechanisms [3,8]. The absence of sampling frames and tracking systems makes it difficult to detect and understand the health and risk patterns of trafficked workers. Also, occupational health research is often based on routinely collected secondary data (official register statistics or surveys based on representative samples of the population), and trafficked persons are seldom if ever in these registers because the workers are inaccessible. These problems are exacerbated in the case of undocumented workers, a hard-to-reach population of unknown size and for whom language and cultural barriers and fear of reprisal complicate the process of conducting quality research. In additional, traditional surveys of working conditions are not designed to take into account the types of extreme working conditions experienced by trafficked workers [8].

Many of the work sectors that are at risk of involvement in labour trafficking have official OHSs. However, trafficked workers generally do not come into contact with these services. Most trafficked workers are temporary workers, and OHSs tend not to provide services for temporary workers. Trafficked workers usually attend health services only when they need immediate or critical assistance. Health professionals at acute wards in hospitals, primary health services, and OHSs should be particularly aware of certain health problems that can be signs of trafficking [9], for instance, acute chemical intoxications, reactions to extreme heat or cold, and accidental traumatic and overuse injuries (Table 1).

Although most people have no access to healthcare while being trafficked, a small proportion of people do come into contact with health providers and therefore can be identified and referred for further services [10].

Health professionals should also take into account the existence of occupational health issues that are specific to trafficked labourers. Trafficked labourers often work longer hours and more days per week than standard workers, leading to high risk of injuries and stress-related problems. Moreover, trafficked labourers may suffer from serious mental health problems as a result of the violent and inhumane treatment they experience, and they might be identified as patients with acute, serious mental disorders or with drug problems [2]. Other long-term health effects of workplaces, including silicosis from dust exposure and reduced hearing due to noise, are less relevant as indicators of trafficking. Many trafficked workers have inadequate nutrition. Trafficked labourers are often not offered health services due to cost, lack of health insurance, and their lack of legal status. Also, continuing to work while ill (presenteeism) is frequent. These workers often live in unsanitary, substandard, and overcrowded houses, and as a result, they have an increased risk of communicable diseases. They frequently go to work with untreated infections [3,9].

## Increasing awareness among all types of health personnel

Health professionals should be able to contribute to the important work of identifying and properly treating trafficked workers, in addition to reporting cases (Table 2). The ILO suggests a set of 11 indicators to identify and assess a forced-labour situation. The indicators include abuse of a worker’s situation of vulnerability, deception, restriction of movement, isolation, physical and sexual violence, intimidation and threats, retention of identity documents, withholding of wages, debt bondage, abusive working and living conditions, and excessive overtime. Sometimes the presence of a single indicator implies forced labour; other times it is necessary to consider several indictors together [11]. This might be observed, for instance, when a worker comes to a hospital with a serious head trauma after falling from a scaffold at a building site. The worker might not have any identification papers, a home address, or provide the name of any employer. This situation is difficult, and health professionals need to be educated in knowing the proper steps to take. Currently, a major problem is that health professionals are often not aware of what trafficking is and have not considered how to handle such a problem. There is a risk of worsening the problem and putting the workers in even greater danger if the situation of trafficking is revealed, so discretion is important when trafficking is suspected. Due to different legislation and official responses, the solutions to these problems vary in different countries, and local strategies and procedures must be developed to properly handle these situations. It is important for the health personnel in all countries to be involved in developing the specific response to these challenges. When response procedures are in place, health personnel should be formally trained on how to handle problems related to trafficking [10]. Training should include provision of information on in-country referral and support options for trafficked people as well as national reporting requirements. Situations involving trafficking victims that have been identified show that the victims involved are unaware of their labour rights and their employers’ obligations on their behalf, such as social security contributions.

**Table 2:** Examples of opportunities for OHSs to address labour trafficking.

General tasks for occupational health personnel	How the task can be a tool to address labour trafficking
Surveillance of the working environment	• Plan workplace visits in work sectors at risk for trafficking, looking for non-registered workers or groups of migrant workers. • Create mechanisms to make unannounced inspections of workplaces possible.
Information to employers and employees	• Provide information about trafficking and health consequences in work sectors at risk; information should also be provided in the language of the workers.
Assessment of health risks in the work environment	• Conduct evaluation of specific risk factors known to be associated with trafficking, including work hours, shift work schedules, or lack of protective equipment.
Surveillance of worker health	• Engage in surveillance of occurrence of injuries in work sectors at risk of trafficking. • Improve follow-up procedures for injured workers. • Develop a short checklist for occupational health personnel with indicators of trafficking.
Advisory role in decision-making processes	• Advise on the inclusion of temporary workers in training and safety information meetings, and also provide information in relevant languages.
Provision of occupational healthcare services	• Provide individual care and/or cure for occupational health problems and make sure consultations are performed in private. • Provide the patients with clear information in their language and perform consultations where gender preferences are followed. • Increase awareness of post-traumatic stress disorder. • Increase awareness of referral pathways for trafficked people, making it easier for them to access healthcare. • Provide holistic care addressing health and social needs.

Abbreviation: OHS, occupational health service

Occupational health personnel have opportunities to be in close contact with workplaces. As mentioned earlier, trafficked people are not likely to contact OHSs. However, occupational health personnel can be proactive in identifying problems. Several activities carried out in OHSs can be useful to identify situations of labour trafficking. Also, OHSs should consider providing services for temporary employed workers (Table 2).

## From challenges to opportunities and solutions

The ILO developed the legally binding Protocol for Forced Labour in 2014 [12], which requires governments to work on the levels of protection, prevention, and compensation in order to address forced labour. Countries must first ratify the Protocol before being subject to it. Governments must implement robust laws and measures that seek to address labour trafficking at its multiple sources. So far, just a few countries have ratified the protocol, but as it gains more widespread recognition and implementation, it will have the potential to promote a coherent institutional response to labour trafficking that includes provisions for protection of victims and prevention of labour trafficking practices.

Information and education about the existence of labour trafficking and how to handle trafficked patients is highly needed among health professionals. Policies and response procedures must be developed in all countries. Other groups, such as migrants, should also be educated about labour trafficking. Targeting these groups can prevent their involvement in forced labour situations, just as raising awareness can help to prevent and encourage the identification of forced labour. Employers must also be educated and informed. Providing employers with information about possible indicators of forced labour may help to prevent these situations from arising. In addition, other actors, such as police, social workers, and labour inspectors, need specific information and education. Legislation and enforcement is also important. Forced labour cases can involve simultaneous violations of labour laws relating to wages, hours of work, occupational safety and health, or other areas. Lastly, victims should be identified, removed from situations of forced labour, and provided with means to recover.

Labour trafficking is a multidisciplinary challenge that, in order to be successfully addressed, requires institutional guidance and cooperation across different geographic regions and administrative disciplines, one of the most important of which is occupational health.

## Abbreviations

ILO: International Labour Organization
OHS: occupational health service
